# Differentiated Thyroid Cancer in Children and Adolescents: Long Term Outcome and Risk Factors for Persistent Disease

**DOI:** 10.3390/cancers13153732

**Published:** 2021-07-24

**Authors:** Giulia Sapuppo, Dana Hartl, Brice Fresneau, Julien Hadoux, Ingrid Breuskin, Eric Baudin, Charlotte Rigaud, Joanne Guerlain, Abir Al Ghuzlan, Sophie Leboulleux, Martin Schlumberger, Livia Lamartina

**Affiliations:** 1 Endocrinology, Department of Clinical and Experimental Medicine, National Centre of Excellence Garibaldi Hospital University of Catania, Garibaldi-Nesima Hospital, 95122 Catania, Italy; giuliasapuppo@hotmail.it; 2 Department of Nuclear Medicine and Endocrine Oncology, Gustave Roussy, 94805 Villejuif, France; julien.hadoux@gustaveroussy.fr (J.H.); eric.baudin@gustaveroussy.fr (E.B.); sophie.leboulleux@gustaveroussy.fr (S.L.); martin.schlumberger@gustaveroussy.fr (M.S.); 3 Departments of Otolaryngology Head and Neck Surgery, Département de Chirurgie, Gustave Roussy, 94805 Villejuif, France; dana.hartl@gustaveroussy.fr (D.H.); ingrid.breuskin@gustaveroussy.fr (I.B.); joanne.guerlain@gustaveroussy.fr (J.G.); 4 Department of Childhood and Adolescent Cancer, Gustave Roussy, 94805 Villejuif, France; brice.fresneau@gustaveroussy.fr (B.F.); charlotte.rigaud@gustaveroussy.fr (C.R.); 5 Department of Medical Biology and Pathology, Gustave Roussy, 94805 Villejuif, France; abir.alghuzlan@gustaveroussy.fr

**Keywords:** thyroid cancer, children, ongoing risk stratification, response to treatment, persistent disease, childhood thyroid cancer

## Abstract

**Simple Summary:**

Despite their excellent prognosis, pediatric differentiated thyroid cancers (P-DTC) often undergo aggressive treatment due to the advanced disease presentation. Reliable risk stratification tools to guide management are needed; unfortunately, the current American Thyroid Association (ATA) classification for P-DTC lacks an unequivocal definition of the three risk categories. In line with previous work, our data confirm a favorable long-term outcome in P-DTC including cases with distant metastases. We propose a modified ATA pediatric risk stratification using a cut-off of five lymph nodes as proposed by the 2015 ATA guidelines for adult DTC. The modified pediatric ATA risk class independently predicted short- and long-term outcome. The utility of applying dynamic risk classification was also confirmed as P-DTC with an excellent response seldom experiences relapse.

**Abstract:**

*Background:* Pediatric differentiated thyroid cancer (P-DTC) frequently presents with advanced disease. The study aim was to evaluate the outcome of P-DTC and a modified 2015 American Thyroid Association risk classification (ATA-R). *Methods:* A retrospective study of consecutive P-DTC patients was performed. The ATA-R for P-DTC was used with a cut-off of ≤ 5 N1a for low-risk. The outcome could be excellent response (ER) (thyroglobulin < 1 ng/mL and no evidence of disease (EoD) at imaging), biochemical incomplete response (BIR) (thyroglobulin ≥ 1 ng/mL and no EoD at imaging) or structural incomplete response (SIR) (EoD at imaging). *Results:* We studied 260 P-DTC (70% females; median age at diagnosis 14 years; 93% total thyroidectomy and 82% lymph node dissection). The ATA-R was low in 30% cases, intermediate in 15% and high in 55%, including 31.5% with distant metastases. Radioiodine treatment was administered in 218 (83.8%), and further radioiodine and surgery was performed in 113 (52%) and 76 (29%) patients, respectively. After a median follow-up of 8.2 years, the outcome was ER in 193 (74.3%), BIR in 17 (6.5%) and SIR in 50 (19.2%). Independent predictors of SIR or BIR at first and last visits were ATA-R intermediate or high. *Conclusion:* P-DTC has an excellent prognosis. Modified ATA-R is a useful prognostic tool in P-DTC to guide management.

## 1. Introduction

Differentiated thyroid cancer (DTC) is rare in childhood. The recent evidence of the frequent presence of clinically silent thyroid cancers in children [1] and the close association of their increased incidence in children/adolescents with that in adults [2] suggests that screening may play an important role in this increased incidence observed in recent years [3,4], although environmental factors might also be involved [5]. 

Compared with DTC in adulthood, children disclose more frequently advanced disease at presentation, including a higher rate of lymph node (LN) and distant metastases (DM) [6,7,8], although screening may lead to the diagnosis of an increased number of patients with localized low-volume disease. The 10-year disease-specific survival rate is almost 100% [9,10,11], and most of the rare cancer-related deaths occur several decades after initial treatment, at an adult age. Despite this excellent outcome, pediatric DTC patients with advanced disease often undergo multiple treatment courses with surgery and radioactive iodine (RAI), with potential morbidity.

Many pediatric patients with iodine-avid microscopic lung metastases can reach a complete structural remission after multiple RAI treatment courses [12,13,14]. Radioiodine-refractory disease is rare in pediatric patients and is rarely rapidly progressive.

Despite the 2015 publication of specific recommendations for the management of thyroid cancer (TC) in children and adolescents by the American Thyroid Association (ATA) [15], their optimal management is still debated due to the lack of high-quality evidence. A three-tiered risk stratification for pediatric DTCs is proposed by the ATA [15] but the definition of the three categories is not unequivocal, rendering its use in clinical practice challenging and it is scarcely reproducible in research studies. 

In the present study, we retrospectively evaluated the early and long-term outcomes in a large consecutive series of pediatric DTC patients treated and followed at Gustave Roussy. We also evaluated the prognostic factors of persistent disease and tested a modified pediatric ATA risk classification. This is an update of our previous report published in 1987 [16].

## 2. Patients and Methods

Consecutive cases of DTC of follicular cell origin diagnosed from 1956 to 2017 were obtained from Gustave Roussy digital archives.

We included all children and adolescents up to 18 years of age at diagnosis, referred to Gustave Roussy for treatment and who were followed up for at least 2 years. Histological review of tumors operated on outside Gustave Roussy was performed by our institutional pathologists.

### 2.1. Initial Post-Operative Classification

Tumors were staged according to the pTNM 8th edition [17]: T (the extent of the primary tumor) and N (regional LN metastases) were assessed based on the pathological examination and M (distant metastases) according to the first post-therapeutic 131I-whole-body scintigraphy (WBS) and/or other imaging modalities (chest X rays and since the 1980s, CT scan). The risk of recurrence was classified according to the American Thyroid Association (ATA) pediatric guidelines [15] as low (intrathyroidal disease, N0 or Nx or incidental finding of a small number of central neck LN metastases (N1a)); intermediate (extensive N1a or minimal lateral (N1b) LN metastases) and high-risk (extensive N1b LN metastases or gross extrathyroidal extension with or without DM). The definition of the extent of LN involvement in the pediatric ATA guidelines does not provide a cut-off for “small” and “extensive” lymph node metastases, and we chose to modify the pediatric ATA risk classification using the criteria proposed by the 2015 ATA guidelines for adults [18]. We considered as low-risk patients those with ≤5, not clinically evident N1a; high-risk patients as those with >5 N1b or any LN metastasis of ≥3 cm or the presence of any clinically detected LN metastases (cN1). The other patients were considered as intermediate-risk. When the number of metastatic LNs was not available (32 cases), the patients were classified according to the other available risk features: 24 patients had distant metastases at 131I-WBS, 1 patient had clinical LNs and 7 patients had ≤5 lymph nodes removed. Cases in which the pathological report did not allow a correct risk classification were excluded.

### 2.2. Treatment

All patients underwent thyroid surgery with or without LN dissection. The indication for RAI treatment was discussed at the institutional multidisciplinary tumor board. RAI treatment activity was empirically 1 mCi/Kg of body weight and was administered following LT4 withdrawal. A 131I-WBS was performed 3–5 days after treatment. 

### 2.3. Response to Initial Therapy

The response to initial therapy was assessed within 6–12 months from initial treatment with serum thyroglobulin (Tg) measurements, either on levothyroxine (LT4) treatment or following TSH stimulation in RAI-treated patients (achieved with LT4 withdrawal or with recombinant human TSH (rhTSH) injections), anti-thyroglobulin antibodies (TgAb) and imaging (diagnostic 131I-WBS with 1–4 mCi and since the 1980s with neck ultrasonography). 

Excellent response (ER) to treatment was defined as an undetectable or a low and stable serum Tg, the absence of TgAb and the absence of abnormal imaging findings. Tg was considered undetectable when it was below 1 ng/mL on LT4 treatment or below 2 ng/mL after TSH stimulation. Disease remission in patients who underwent a lobectomy was defined as stable Tg and the absence of abnormal findings on neck ultrasound. Persistent disease was considered as biochemically incomplete response (BIR) in the absence of abnormal findings on imaging with either detectable serum Tg in patients treated with RAI, rising Tg values over time in patients treated or not with RAI, or the presence of serum TgAb. Structurally incomplete response (SIR) was defined as the presence of abnormal findings on imaging. Progressive structural disease was defined as an increase in the number or the volume of metastatic lesions according to RECIST criteria [19].

In patients with persistent/recurrent disease, additional morphological examinations such as computed tomography (CT), magnetic resonance imaging (MRI) and 18 fluorodesoxyglucose positron emission tomography (FDG-PET) were performed as required, and further treatments (RAI therapy, surgery or other therapies) were administered. 

The serum TSH goal could vary over time, and usually, it was adapted to the initial response to treatment: in patients at low risk and in ER, TSH was maintained in the low–normal range; in patients at high risk or with persistent biochemical or structural disease, the TSH goal was <0.1 uUI/mL with normal free T4.

### 2.4. Thyroglobulin Assay 

The serum Tg assay became available in 1977 using a homemade radio-immuno assay (RIA) with a functional sensitivity of 1.6 ng/mL [20]. From 1990 until 2005, an IRMA method (Medipan, functional sensitivity of 1 ng/mL) was used. From 2006 onwards, serum Tg level was assessed with a chemiluminescent immunoenzymatic assay (Access assay, functional sensitivity of 0.1 ng/mL). The Tg level was considered as not accurately measured when the recovery test was less than 80% and since 2006 in the presence of TgAb with the Access Thyroglobulin Antibody II assay [21]. 

### 2.5. Statistical Analysis

Categorical variables were expressed as frequencies and percentages and analyzed using the Chi-square test with Yates’ correction or Fisher’s test for small samples. Normally distributed quantitative variables were expressed as mean ± standard deviation (SD), while non-normally distributed variables were expressed as median and interquartile range (IQR). Quantitative variables were analyzed by the Student’s *t*-test or the Mann–Whitney U test. Multiple logistic regression analysis was performed for all variables having significant results at univariate analysis to identify independent risk factors associated with persistent/recurrent disease; some variables with a high rate of missing data were excluded. A *p* value < 0.05 was considered statistically significant for all analyses. Data analysis was performed using the SPSS software version 13.0 (IBM Corp, Armonk, NY, USA).

Data were collected in an anonymized electronic file. The study was approved by the Gustave Roussy Ethics Committee and the legal tutors of all the patients signed a written consent form for the participation in observational research.

## 3. Results

### 3.1. Characteristics of the Patients

The characteristics of the 260 patients included in the present study are shown in Table 1. Most patients were female (70.4%), the median age at diagnosis was 14.2 yrs (IQR 11.1–16.3). Previous radiation exposure to the neck was reported in 13 patients and a family history of follicular cell-derived TC in 13 patients.

Total thyroidectomy was performed in 241 patients (92.7%). LN surgery was performed in 214 (82.3%) patients, central compartment LN dissection in 71 and, central and lateral LN dissection in 143 patients. 

Histotype was papillary in 206 (79.2%) cases, of which 26 were a diffuse sclerosing variant and 16 a solid variant, and a follicular carcinoma was found in 33 (12.7%) cases.

Lymph node metastases were found in 82.5% of patients who underwent lymph node dissection. Among N1 patients, the number of lymph node metastases ranged from one to 59 (median 10). No significant difference in terms of persistent disease was found comparing N0 or Nx with N1 cases having ≤5 LN metastases both for early and long-term outcome assessments, supporting the inclusion of these patients in the low-risk category, as we did throughout the present study. In contrast, the risk of persistent disease was significantly higher for patients with more than five LN metastases, of the central (N1a) or of the lateral (N1b) compartment.

Eighty-two (31.5%) patients had DM at diagnosis (lung in 78 cases, lung and bone in three and, lung and brain in one). 

After initial treatment, patients were classified as low-risk (77, 29.6%), intermediate-risk (41, 15.8%) and high-risk (142, 54.6%) of recurrent/persistent disease (Table 1). The rate of SIR, M and ATA risk class did not show significant modification over time (Figure S1).

### 3.2. Postoperative RAI Administration

RAI treatment was administered in 218 (83.8%) patients; repeated administrations were needed in 113 (52.3%) patients (two–sixteen treatments). The median cumulative activity (med-131I) administered to these 218 patients was 200 mCi (range 30–1040 mCi) and in the 82 patients with DM it was 324 mCi (range 60–1040 mCi). 

Of the 42 patients not treated with RAI, 25 were low-risk, seven intermediate-risk and 10 high-risk patients. The intermediate- and high-risk patients not treated with RAI were diagnosed before 1996. At the last visit, 40 of these 42 patients were cured (95.2%), one initially low-risk patient had persistent LN metastases and one initially high-risk patient died from local and distant progressive disease.

One patient developed leukemia after a cumulative activity of 220 mCi and one patient developed pulmonary fibrosis after a cumulative activity of 810 mCi.

### 3.3. Response to Initial Therapy, 6–12 Months after Initial Treatment and at Last Follow-Up

The median follow-up was 8.2 years (range 2–61 yrs). 

**Figure 1:**
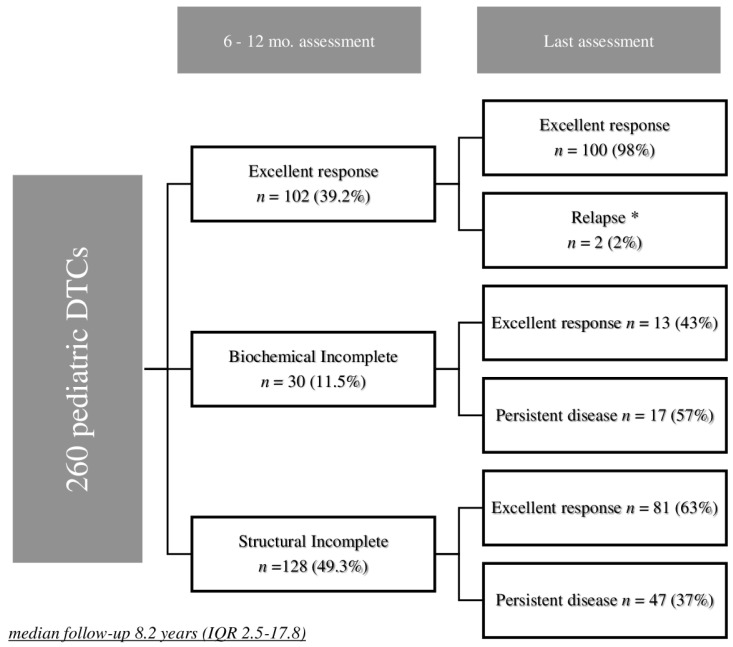
Outcome at 6–12 months after initial treatment and at last follow-up in 260 pediatric DTCs. * A total of 1 BIR (appearance of TgAb) needing a second RAI with a negative WBS had ER at last visit; 1 SIR (metastatic LN on ultrasound) needing neck surgery and RAI had SIR (small LN) at the last visit.

At 6–12 months after initial treatment, 102/260 (39.2%) patients presented with ER, 30 (11.5%) with BIR and 128 (49.3%) with SIR (48 persistent N1 and 80 DM) (Figure 1). 

ER after initial treatment was observed in 80% of low-risk patients, 46.3% of intermediate-risk and 15.5% of high-risk patients (*p <* 0.01). Among these 102 patients with ER, only two patients initially classified as intermediate-risk had a recurrence 7 and 8 years after the initial treatment. 

Among the 30 patients with BIR, three showed a spontaneous disappearance of TgAb after 4 years, 21 had further RAI treatment courses (11 a second RAI treatment course and 10 had three or more RAI treatments) and post-therapy 131I-WBS revealed foci of uptake in LN areas (all in the neck or mediastinum) in 16 patients; nine patients had further neck surgery in addition to further RAI treatment courses (with histological confirmation of DTC in all cases). At last follow-up, ER was achieved in 13/30 (43.3%) patients with initial BIR, seven had persistent BIR and 10 SIR (three LN metastases and seven DM). 

Among the 128 patients with SIR after the initial treatment, 108 had further RAI with a med-131I of 215 mCi (IQR 195–412 mCi). Sixty patients underwent further surgery for LN metastases; 11 had three surgical treatments and two had four surgeries. Five patients were treated with neck and mediastinal external beam radiotherapy. Two patients were treated with cytotoxic chemotherapy and one with tyrosine kinase inhibitors. At last follow-up, ER was achieved in 81/128 (63.3%) patients with initial SIR. 

Of the 82 patients with initial DM, in 76 DM was detected at 131I-WBS, in five through a CT scan and in one through a chest X-ray. Forty-three (52%) patients were in ER at last control and 39 (48%) had persistent disease (four BIR and 35 SIR including metastatic neck LNs in five, DM in 26 and both neck LNs and DM in four). DM patients with a follow-up longer than 5 years had a higher rate of ER at their last visit: 8/23 (35%) if the follow-up was <5 years; 10/16 (63%) if 5–10 years and 24/42 (57%) if >10 years. 

Independent predictors of the presence of DM at diagnosis were larger tumor size, multifocality and N1b (Table 2).

Overall, at last follow-up, 193/260 (74.3%) patients were ER, 17 (6.5%) BIR (15 detectable Tg and two positive TgAb) and 50 (19.2%) SIR (15 LN metastases, 29 DM and six both LN and DM). Interestingly, 12 of the 16 (75%) low-risk patients with BIR and SIR after initial treatment were cured at the last follow-up visit, albeit after multiple RAI treatment courses or surgeries.

Fourteen SIR patients (5%) were considered RAI refractory: 10 patients had no RAI uptake in one or more metastatic sites at first or further post therapeutic WBS (four patients experienced disease progression), one patient progressed despite RAI uptake and three patients were not cured despite an administered cumulative RAI activity of 600 mCi or more. 

Overall, 64 patients (24.6%) were not cured at the last visit (BIR+SIR): they represent 5.2% of the low-risk, 31.7% of intermediate-risk and 34.6% of high-risk patients.

Only 9 (19%) of SIR patients had morphologic disease progression, i.e., 3.5% of all cases and five patients died during the follow-up—two from unrelated causes (one from Bourneville syndrome and one from an accidental drowning) and three from DM of TC at an age of 45, 34 and 16 years (Table 3). 

### 3.4. Surgical Reoperations and Complication Rates

Almost a third of the patients (75/260, 28.8%) required more than one surgical procedure, in addition to a greater number of RAI treatment courses (med-131I 260 mCi, range 40–1040). Only 1/39 (2.5%) N0 patients at primary surgery had neck re-operation for LN recurrence compared with 15/46 (32.6%) Nx (*p* < 0.001), 5/32 (15.6%) N1a (*p* = 0.08) and 54/143 (37.8%) N1b patients (*p* < 0.001). The rate of reoperation was similar in Nx (15/46, 32.6%) and N1 patients (59/175, 33.7%).

At last follow-up, 27 (41.5%) of the 75 re-operated patients were not cured, including 21/64 who had two surgeries (7 BIR and 14 SIR, 5 LN persistence, 6 DM and 3 both), and 6/11 who had three or four surgeries (2 BIR and 4 SIR, LN persistence).

The overall rate of complications after primary ± re-operative surgery was 20%: 10.8% permanent hypoparathyroidism (28 patients), 4.6% unilateral permanent recurrent laryngeal nerve palsy (RLN) (12 patients, of which two underwent RLN sacrifice due to tumor invasion), 4.6% other or multiple complications (12 patients, of which one underwent RLN sacrifice due to tumor invasion). The rate of complications on patients operated at Gustave Roussy, excluding cases who needed voluntary nerve sacrifice, was 7.6%: hypoparathyroidism 2.7%, permanent RLN 1.9%, other and multiple complications 3%. 

The complication rate was comparable based on age at diagnosis (22.9% if <10 years old and 19.3% if ≥10 years old, *p* = 0.57), on the extent of surgery (18.1% if thyroidectomy alone and 23.9% if thyroidectomy + neck dissection, *p* = 0.27) and on the number of surgical procedures (22.2% if one procedure and 12.3% if two procedures, *p* = 0.09). A higher rate of complications was observed in patients who underwent LN reoperation: 37.5% (24/64) after the first reoperation and 63.6% (7/11) after the second and third reoperation, however this was not statistically significant (*p* = 0.1).

### 3.5. Predictors of Early and Late Persistent Disease 

Risk factors for persistent disease (BIR or SIR) at 6–12 months after primary treatment and at the last visit are presented in Table 4 and Table 5, respectively.

Although the distribution in risk categories was different based on age at diagnosis with a higher percentage of high-risk patients among younger patients, no significant difference in outcome (at 6–12 months after initial treatment and at last visit) was found according to the age at diagnosis.

In multivariate analysis, independent predictors of persistent disease at 6–12 months were multifocality and ATA intermediate- and high-risk (Table 4) and at last visit, ATA intermediate- or high-risk (Table 5).

Analyzing only structural persistent disease at the last follow-up (Table 6), ATA intermediate- or high-risk were confirmed as independent predictors.

## 4. Discussion

Compared to DTC in adulthood, children show different clinical, pathological and molecular characteristics [22], with a higher rate of LN (50–75%) and DM (6–33%) [8,23,24,25]. Our findings are consistent with previous reports, with a rate of LN metastases and DM at presentation of 67% and 31%, respectively. 

In the pediatric population, no single postoperative staging system has been validated yet. The TNM classification is the most frequently used classification to define the risk of mortality but with the limitation that all pediatric patients without DM fall into stage I. The risk classification proposed by the ATA for pediatric patients is a valuable tool to guide initial management, but its definition is not unequivocal. We used a modified pediatric ATA risk classification adopting a threshold of ≤5 N1 as proposed by the adult ATA risk classification [18] to define a “small” number of metastatic LNs. Indeed, we found no significant difference in terms of persistent disease comparing N0/Nx cases with N1 cases having ≤5 LN metastases both for early and long-term follow-up, supporting the inclusion of these patients in the low-risk category. The modified pediatric ATA risk classification was an independent predictor of the early and late outcomes in our series, supporting its use in the management of pediatric DTC patients. Younger age at diagnosis was associated with a more extended presentation, as already reported [26,27], but the outcome was not related to the age at presentation. 

Extensive disease presentation mirrors the frequent use of extensive surgery and RAI. This aggressive therapeutic approach might be justified by the rarity of recurrent disease in patients who achieved an excellent response, in accordance with other studies [9]. In our study, about 50% of patients had two or more RAI treatments, a higher percentage compared with other literature reports [9]. This is probably due to a selection bias, as Gustave Roussy is a national and international reference center that concentrates patients with more advanced disease stages needing repeated treatments and also to the frequent use of RAI-guided surgery. Interestingly, the rate of patients with SIR and the rate of patients with M1 did not significantly change over the past decades (Figure S1). 

As compared to our previous report in 1987, external beam radiotherapy was seldom used for the treatment of pediatric DTCs, namely in most recent years, probably due to the evolution of diagnostic and therapeutic tools. 

The risk of LN recurrence is low in N0 patients, and the benefits of surgery in such patients are to permit a reliable prognostic classification and possibly to avoid post-operative RAI administration in some. However, the risk of LN recurrence remains high in N1 patients, being higher in patients with >5 N1 and in N1b than in N1a patients probably due to more extensive and aggressive disease. With regards to the high morbidity of surgical procedures, the indication of prophylactic LN dissection, in those patients without LN involvement detected clinically or at neck ultrasound, should be carefully discussed in terms of risks and benefits. 

Despite the extensive disease at presentation, pediatric patients have an almost 100% 10-year disease-specific survival [8,9,10,11,24]. 

DM in pediatric patients usually presents as micronodular, radioiodine-avid diffuse lung lesions and more than half of these patients reached an ER after multiple RAI treatment courses, in agreement with our previous reports [12,13,14,28]. However, these high rates of ER were achieved after repeated treatments, including the administration of high cumulative activity of 131I (mean of 324 mCi) and repeated surgery performed in roughly half of the cases. Even in the case of persistent disease refractory to RAI, it was stable or very slowly progressive and only 19% of our SIR patients (i.e., 3.5% of all cases) had structural progressive disease and three (1.2%) patients died from TC, two from lung metastases and one from lung and brain disease.

Despite the wide consistency of low mortality rates in pediatric DTCs across different studies, discordant rates of persistent disease are found in the literature according to the population studied.

A report from a US referral center on 148 pediatric DTC with DM found a persistent disease rate of 93% after a median follow up of 10.5 years [29], and this striking difference compared to our results, despite a similar median administered cumulative activities of radioiodine, may rely on different evaluation criteria used for treatment response. We have previously shown that in adult patients who had no residual imaging abnormalities after RAI treatment for DM, the residual Tg level (detectable or not) had no predictive role on subsequent progression [16]. This is confirmed by the ATA guidelines: “an undetectable Tg level should no longer be the sole goal of treatment of children with pulmonary metastases” [15]. Furthermore, persistent lung micronodules without evidence of residual RAI uptake might be related to either persistent disease or post-radiation sclerosis.

On the other hand, the percentage of BIR and SIR at the last follow-up in our study (25%) was higher than that reported in other series [9,26], possibly due to more extensive disease related to a referral center bias, but this is also in accordance with the use of sensitive tools for detecting disease. 

Regarding the risk factors of persistent disease, our data confirm that multifocality [30,31] and ATA class independently predicted short- and long-term outcome.

The use of dynamic risk classification [18] in pediatric and adolescents is supported by our study: patients with an excellent response to initial treatment have a low risk of recurrence, with only 2/102 patients initially classified as intermediate-risk (1.9%) experiencing a recurrence. Therefore, follow-up might be less intensive for these patients. In two previous publications, the risk of recurrence of the patients in ER was higher (13–30%) [26,32], and this difference is possibly due to different methods used to classify patients in ER. The probability of achieving ER after initial treatment depended on risk category (from 80% in low-risk to only 15.5% in high-risk patients). 

Only 10% (n = 3) of patients with BIR at the first assessment achieved ER without further treatment, and this rate might possibly be higher if one would consider biological abnormalities without abnormal findings on imaging as a non-actionable finding, as advocated by the ATA recommendations [15], and BIR might be a reasonable objective for some patients in the absence of demonstrated survival benefit and in order to avoid possible complications related to further treatments. 

Our study has several limitations, including the potential selection bias of a single referral institution, the long recruitment period (longest follow-up of 61 years), the lack of a molecular characterization of these tumours and its retrospective nature. However, few patients were lost to follow-up.

## 5. Conclusions

In conclusion, our data confirm that in pediatric and adolescent patients with DTC, despite the aggressive initial presentation, the long-term outcome is favorable. Even patients with BIR and SIR generally have non-progressive disease and long survival, and mortality from TC is low. The modified ATA risk classification, incorporating criteria used in adults for LN metastases and the dynamic risk classification, are valuable tools to guide the management of pediatric and adolescent DTC patients. An effort should be made to reduce the treatment burden and morbidity rates in these patients. 

## Figures and Tables

**Table 1:** Clinical and histopathological characteristics of the 260 pediatric DTC patients aged ≤18 years old (y/o) diagnosed between 1956 and 2017.

Variation	*n*	(%)
Patients (*n*.)	260	
Follow-up median (IQR) (y) *	8.2 (2.5–17.8)	
Median age (IQR) (y)	14.2 (11.1–16.3)	
Gender		
F/M (ratio)	183/77 (2.4/1)	
F/M <10 y/o	27/21 (1.3/1)	
F/M >10 y/o	156/56 (2.8/1)	
Histotype		
Papillary	217	83.5
Follicular	33	12.7
Not known	5	1.9
Poorly differentiated	4	1.6
Uncertain malignancy potential	1	0.4
TNM (VIII ed.)		
T status (T)		
T1a	31	11.9
T1b	43	16.5
T2	74	28.5
T3a	40	15.4
T3b	10	3.8
T4	8	3.1
Tx	54	20.8
N status (N)		
N0	37	14.2
N1a	32	12.3
N1b	143	55.0
Nx/N0b	48	18.5
M1	82	31.5
Extra thyroidal invasion		
No	176	67.7
minimal	79	30.4
gross	5	1.9
Multifocal tumor	102	39.2
Thyroglossal cyst duct carcinoma	4	1.6
Modified pediatric ATA risk of recurrence categories		
Low	77	29.6
Intermediate	41	15.8
High **	142	54.6

* Follow-up minimum 2 years and maximum 61.3 yrs ** (M1, N1b Ln > 3 cm and > 5 and cN1).

**Table 2:** Risk factors for the presence of distant metastases at diagnosis.

Variable	M1/All Patients 82/260	Univariate Analysis OR (95%CI)	*p*	Multivariate Analysis OR (95%CI)		*p*
Age			0.04	0.59 (0.29–1.22)		0.16
<10 years	21/48 (43.8%)	1.93 (1.01–3.66)				
≥10 years	61/212 (28.8%)	0.52 (0.27–0.99)				
Gender						
Female	59/183					
Male	23/77	0.9 (0.50–1.60)	0.70			
Lymph node surgery at primary treatment						
Performed	70/214 (32.7%)					
Not performed	12/46 (26.1%)	0.73 (0.35–1.49)	0.38			
Aggressive histology						
No	65/205 (31.7%)					
Yes	17/55 (30.9%)	0.96 (0.51–1.83)	0.90			
Tumor size						
<1 cm	2/29 (6.9%)					
1-≤2 cm	12/45 (26.7%)	4.91 (1.01–23.86)	0.03	3.72 (0.72–19.08)		0.12
2-≤4 cm	26/82 (31.7%)	6.27 (1.38–28.37)	0.008	6.91 (1.41–33.85)		0.02
>4 cm	13/38 (34.2%)	7.02 (1.44–34.25)	0.008	6.85 (1.26–37.05)		0.03
Unknown	29/66 (43.9%)	7.83 (1.73–35.35)	0.002	14.28 (2.86–71.39)		0.001
Multifocal						
No	38/158 (24.1%)					
Yes	44/102 (43.1%)	2.4 (1.40–4.09)	0.001	2.66 (1.39–5.11)		0.003
Extra thyroidal invasion						
No	49/176 (27.8%)					
minimal	29/79 (36.7%)	1.50 (0.86–2.64)	0.16	1.01 (0.53–2.15)		0.85
massive	4/5 (80%)	10.37 (1.13–95.07)	0.01	5.30 (0.50–55.95)		0.16
Lymph node metastases						
-Absent	13/85 (15.3%)					
-Present	69/175 (39.4%)	3.61 (1.86–7.00)	0.0001	Not included		
Number of N1 at primary surgery						
N0/Nx	13/85 (15.3%)					
≤5 N1	9/46 (19.6%)	1.35 (0.53–3.44)	0.53			
>5 N1	36/97 (37.1%)	3.27 (1.59–6.72)	0.0009			
not known	24/32 (75%)	16.62 (6.15–44.92)	0.0000	Not included		
Location of N1 at primary surgery						
N0/Nx	13/85 (15.3%)					
N1a	6/32 (18.8%)	1.28 (0.44–3.71)	0.65		1.44 (0.45–4.55)	0.54
N1b	63/143 (44.1%)	4.36 (2.22–8.58)	0.000		3.35 (1.61–6.96)	0.0012
Radioiodine treatment						
Not performed	1/42 (2.4%)					
Performed	81/219 (37.0%)	23.48 (3.17–174.04)	0.0000		Not included	

**Table 3:** Three patients died from thyroid cancer.

Patients	Age at Diagnosis	Age at Death	TNM	Histotype	Initial Response	Treatment Performed	Site of Distant Metastases
1	14	45	Tx N1b	PTC *	Persistent disease	TT ° + bilateral LN°° dissection, RTE, 8 ^131^I treatment (820 mCi)	Lung and brain
2	6	34	T4 Nx	FTC **	Persistent disease	TT ° + unilateral LN°° dissection, RTE, 3 ^131^I treatment, chemotherapy	Lung
3	8	16	Tx Nx	PTC *	Persistent disease	TT °, chemotherapy	Lung

* PTC papillary thyroid cancer; ** FTC follicular thyroid cancer; ° TT total thyroidectomy; °° LN lymph node.

**Table 4:** Risk factors of persistent disease at 6–12 months after initial treatment.

Variable	BIR + SIR/All Patients	Univariate Analysis		Multivariate Analysis	
	151/260	OR [95%CI]	*p*	OR [95%CI]	*p*
Age					
<10 years	31/48 (64.6%)				
≥10 years	120/212 (56.6%)	1.3 (0.73–2.68)	0.311		
Gender					
Female	101/183 (55.2%)				
Male	50/77 (64.9%)	1.5 0.87–2.61)	0.146		
Primary surgery					
Total thyroidectomy	145/241 (60.2%)			Not included	
Less than total thyroidectomy	6/19 (31.6%)	3.27 (1.20–8.9110.82)	0.015		
Lymph node surgery at primary treatment					
Performed	130/214 (60.7%)				
Not performed	21/46 (45.7%)	1.84 (0.97–3.5)	0.058		
Intention of initial LN surgery					
Not performed	18/46 (39.1%)				
Prophylactic	27/72 (37.5%)	0.93 (0.44–2.00)	0.85	Not included	
Therapeutic	42/48 (87.5%)	10.89 (3.85–30.82)	0		
Unknown	64/94 (68.1%)	3.32 (1.59–6.91)	0.001		
Aggressive histology					
No	113/205 (55.1%)				
Yes	38/55 (69.1%)	1.82 (0.96–3.43)	0.06		
Tumor size					
<1 cm	12/29 (41.4%)				
1-≤2 cm	27/45 (60.0%)	2.13 (0.82–5.49)	0.11	1.33 (0.37–4.70)	0.66
2-≤4 cm	38/82 (46.3%)	1.22 (0.52–2.88)	0.64	0.88 (0.28–2.74)	0.82
>4 cm	27/38 (71.1%)	3.48 (1.26–9.63)	0.01	3.06 (0.80–11.66)	0.1
Unknown	47/66 (71.2%)	3.50 (1.41–8.72)	0.005	2.27 (0.65–7.94)	0.2
Multifocality					
No	75/158 (47.5%)				
Yes	76/102 (74.5%)	3.23 (1.88–5.57)	0	2.78 (1.355.73)	0.006
Extra thyroidal extension					
No	96/176 (54.5%)			Not included	
minimal	50/79 (63.3%)	1.44 (0.83–2.48)	0.19		
gross	5/5 (100%)	-	0.07		
Lymph node metastases					
Absent	24/85 (28.2%)	6.72 (3.78–11.98)	0		
Present	127/175 (72.6%)				
Number of N1 at primary surgery					
N0/Nx	24/85 (28.2%)				
≤5 N1	20/46 (43.5%)	1.96 (0.92–4.14)	0.13	Not included	
>5 N1	78/97 (80.4%)	10.43 (5.24–20.78)	0		
not known	29/32 (90.6%)	24.57 (6.84–88.29)	0		
Location of N1 at primary surgery					
N0/Nx	24/85 (28.2%)				
N1a	11/32 (34.4%)	1.33 (0.56–3.17)	0.51	0.14 (0.13–1.47)	0.1
N1b	116/143 (81.11%)	10.92 (5.81–20.53)	0	0.36 (0.04–2.96)	0.34
Distant metastases at primary surgery				Not included	
No	69/178 (38.8%)				
Yes	82/82 (100%)	–	0.001		
Modified pediatric ATA risk stratification					
Low	11/77 (14.3%)				
Intermediate	21/41 (51.2%)	6.30 (2.60–15.26)	0	25.09 (2.39–263.66)	0.007
High	119/142 (83.8%)	31.04 (14.25–67.65)	0	66.71 (7.68–579.89)	<0.001
Radioiodine treatment					
Not performed	5/42 (12.2%)			Not included	
Performed	146/219 (66.7%)	14.40 (5.42–38.24)	0		

Abbreviations: BIR: biochemical incomplete response; Clinical disease: presence of clinical findings due to thyroid cancer (palpable lymph nodes, dysphonia, dysphagia); N1: lymph node metastases, SIR: structural incomplete response.

**Table 5:** Risk factors of persistent or recurrent disease at last assessment.

Variable	BIR + SIR/All Patients 66/260	Univariate Analysis OR [95%CI]	*p*	Multivariate Analysis OR [95%CI]	*p*
Age					
<10 years	10/48 (20.8%)				
≥10 years	56/212 (26.4%)	1.36 (0.64–2.92)	0.422		
Gender					
Female	44/183 (24.0%)				
Male	22/77 (28.6%)	1.26 (0.69–2.30)	0.443		
Lymph node surgery at primary treatment					
Performed	55/214 (25.7%)				
Not performed	11/46 (23.9%)	1.10 (0.52–2.32)	0.8		
Intention of initial lymph node surgery					
not performed	8/46 (17.4%)				
Prophylactic	10/72 (13.9%)	0.77 (0.28–2.11)	0.61		
Therapeutic	15/48 (31.3%)	2.16 (0.81–5.73)	0.12		
Unknown	33/94 (35.1%)	2.57 (1.07–6.15)	0.03		
Aggressive histology					
No	49/205 (23.9%)				
Yes	17/55 (30.9%)	1.42 (0.74–2.74)	0.28		
Tumor size					
<1 cm	7/29 (24.1%)				
1–≤2 cm	6/45 (13.3%)	0.48 (0.14–1.62)	0.12		
2–≤4 cm	17/82 (20.7%)	082 (0.30–2.24)	0.7		
>4 cm	15/38 (39.5%)	2.05 (0.70–5.98)	0.18		
Unknown	21/66 (31.8%)	1.47 (0.54–3.97)	0.44		
Multifocality					
No	30/158 (60.8%)				
Yes	36/102 (35.3%)	2.33 (1.32–4.11)	0.003	1.73 (0.95–3.15)	0.07
Extra thyroidal extension					
No	38/176 (21.6%)				
minimal	24/79 (30.4%)	1.58 (0.87–2.88)	0.13	Not included	
gross	4/5 (80%)	14.53 (1.58–133.82)	0.002		
Lymph node metastases					
Absent	11/85 (12.9%)			Not included	
Present	55/175 (31.4%)	3.08 (1.52–6.27)	0.001		
Number of N1 at primary surgery					
N0/Nx	11/85 (12.9%)				
≤5 N1	10/46 (21.7%)	1.87 (0.73.4.81)	0.19		
>5 N1	32/97 (32.9%)	3.31 (1.55–7.09)	0.002		
not known	13/32 (32.9%)	4.60 (1.78–11.88)	0.001		
Location of N1 at primary surgery					
N0/Nx	11/85 (12.9%)				
N1a	11/32 (34.4%)	3.52 (1.34–9.26)	0.008		
N1b	44/143 (30.8%)	2.99 (1.45–6.18)	0.002		
Distant metastases at primary surgery					
No	27/178 (15.2%)			Not included	
Yes	39/82 (47.6%)	5.07 (2.79–9.21)	0		
Modified pediatric ATA risk stratification					
Low	4/77 (5.2%)				
Intermediate	13/41 (31.7%)	8.47 (2.55–28.20)	0	7.75 (2.31–25.97)	<0.001
High	49/142 (34.5%)	9.62 (3.32–27.87)	0	8.32 (2.84–24.42)	<0.001
Radioiodine treatment					
Not performed	1/42 (2.4%)				
Performed	65/219 (29.7%)	16.88 (2.27–125.43)	0	Not included	

Abbreviations: BIR: biochemical incomplete response; Clinical disease: presence of clinical findings due to thyroid cancer (palpable lymph nodes, dysphonia, dysphagia); N1: lymph node metastases, SIR: structural incomplete response.

**Table 6:** Risk factors of structural incomplete response (SIR) disease at last assessment.

Variable	SIR/All Patients 50/260	Univariate Analysis OR [95%CI]	*p*	Multivariate Analysis OR [95%CI]	*p*
Age					
<10 years	8/48 (16.7%)				
≥10 years	42/212 (19.8%)	0.93 (0.40–2.17)	0.86		
Gender					
Female	35/183 (19.1%)				
Male	15/77 (19.5%)	1.02 (0.52–2.01)	0.95		
Lymph node surgery at primary treatment					
Performed	40/214 (18.7%)				
Not performed	10/46 (21.7%)	1.21 (0.55–2.64)	0.63		
Intention of initial lymph node surgery					
not performed	10/46 (21.7%)				
Prophylactic	7/72 (9.7%)	0.39 (0.14–1.11)	0.07		
Therapeutic	11/48 (22.9%)	1.07 (0.41–2.82)	0.89		
Unknown	22/94 (23.4%)	1.10 (0.47–2.57)	0.83		
Aggressive histology					
No	39/205 (19.0%)				
Yes	11/55 (20.0%)	1.06 (0.50–2.25)	0.87		
Tumor size					
<1 cm	4/29 (13.8%)				
1≤2 cm	3/45 (6.7%)	0.45 (0.09–2.16)	0.31	Not included	
2≤4 cm	13/82 (15.9%)	1.18 (0.35–3.95)	0.79		
>4 cm	12/38 (5.3%)	2.88 (0.82–10.15)	0.09		
Unknown	18/66 (27.3%)	2.34 (0.72–7.68)	0.15		
Multifocality					
No	23/158 (14.6%)				
Yes	27/102 (26.5%)	2.11 (1.13–3.94)	0.017	1.56 (0.81–2.98)	0.18
Extra thyroidal extension					
No	28/176 (15.9%)				
minimal	18/79 (22.8%)	1.56 (0.80–3.03)	0.19	Not included	
ross	4/5 (80%)	21.14 (2.28–196.28)	0.0002		
Lymph node metastases					
Absent	10/85 (13.3%)			Not included	
Present	40/175 (22.9%)	2.22 (1.05–4.70)	0.03		
Number of N1 at primary surgery					
N0/Nx	10/85 (13.3%)				
≤5 N1	6/46 (20.9%)	0.60 (0.21–1.73)	0.34		
>5 N1	24/97 (32.9%)	2.47 (1.10–5.52)	0.025		
not known	10/32 (40.6%)	3.41 (1.26–9.24)	0.01		
Location of N1 at primary surgery					
N0/Nx	10/85 (13.3%)				
N1a	7/32 (21.9%)	2.10 (0.72–6.10)	0.17		
N1b	33/143 (23.1%)	2.25 (1.05–4.84)	0.03		
Distant metastases at primary surgery					
No	15/178 (8.4%)			Not included	
Yes	35/82 (42.7%)	8.09 (4.07–16.08)	0		
Modified pediatric ATA risk stratification					
Low	3/77 (3.9%)				
Intermediate	8/41 (19.5%)	5.98 (1.49–23.98)	0.006	5.53 (1.37–22.32)	0.016
High	39/142 (23.9%)	9.34 (2.78–31.37)	0	8.28 (2.43–28.17)	<0.001
Radioiodine treatment					
Not performed	1/42 (2.4%)			Not included	
Performed	49/219 (29.7%)	11.53 (1.55–86.02)	0.003		

Abbreviations: BIR: biochemical incomplete response; Clinical disease: presence of clinical findings due to TC (palpable LN, dysphonia, dysphagia); N1: LN metastases, SIR: structural incomplete response.

## Data Availability

Data are available in the Gustave Institute archive.

## Supplementary Materials

The following are available online at https://www.mdpi.com/article/10.3390/cancers13153732/s1, Figure S1: Rate of structural incomplete response (SIR), distant metastases (M1) and ATA risk classes over time per decade
