# Supplementary material for: Differentiated Thyroid Cancer in Children and Adolescents: Long Term Outcome and Risk Factors for Persistent Disease
Source: Cancers (Basel). 2021 Jul 24;13(15):3732. doi: 10.3390/cancers13153732 (PMC8345030; doi:10.3390/cancers13153732)
Supplement: Supplementary file 1 [file cancers-13-03732-s001.zip › cancers-1300931-supplementary.pdf]

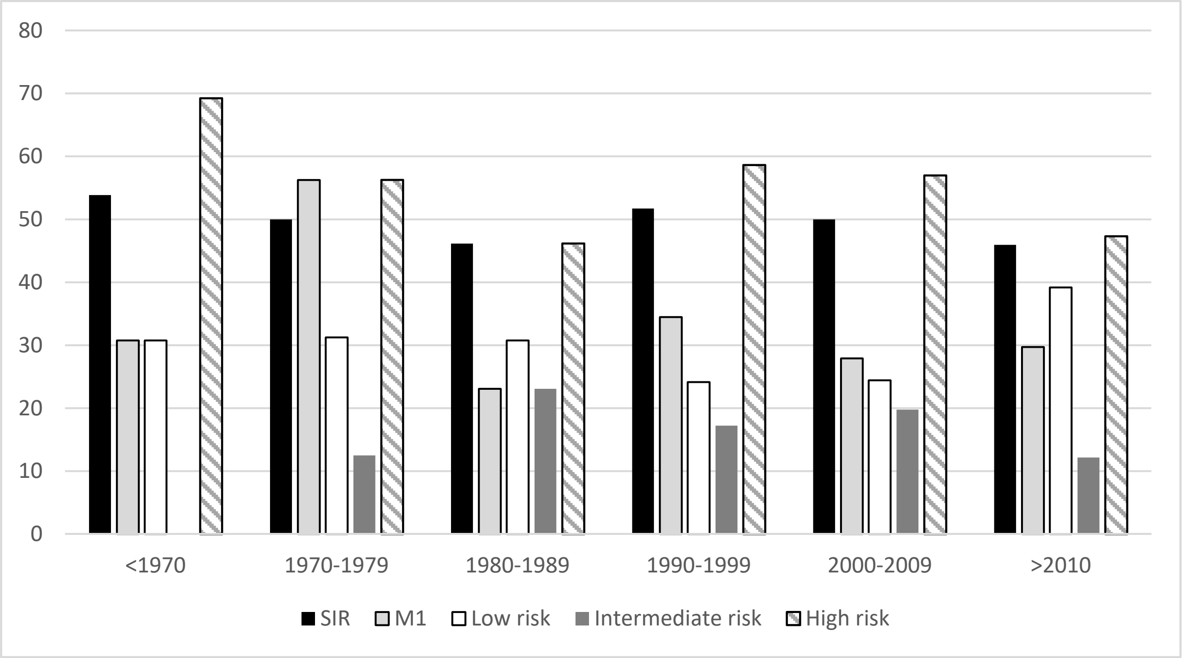

Figure S1: Rate of structural incomplete response (SIR), distant metastases (M1) and ATA risk classes over time per decade.
